# RNA-seq reveals insights into molecular mechanisms of metabolic restoration via tryptophan supplementation in low birth weight piglet model

**DOI:** 10.1093/jas/skac156

**Published:** 2022-05-11

**Authors:** Ping Xiao, Parniyan Goodarzi, Adel Pezeshki, Darren E Hagen

**Affiliations:** Department of Animal and Food Sciences, Oklahoma State University, Stillwater, OK 74078, USA; Department of Animal and Food Sciences, Oklahoma State University, Stillwater, OK 74078, USA; Department of Animal and Food Sciences, Oklahoma State University, Stillwater, OK 74078, USA; Department of Animal and Food Sciences, Oklahoma State University, Stillwater, OK 74078, USA

**Keywords:** lipid metabolism, low birth weight, piglets, RNA sequencing, tryptophan

## Abstract

Low birth weight (LBW) is associated with metabolic disorders in early life. While dietary l-tryptophan (Trp) can ameliorate postprandial plasma triglycerides (TG) disposal in LBW piglets, the genetic and biological basis underlying Trp-caused alterations in lipid metabolism is poorly understood. In this study, we collected 24 liver samples from 1-mo-old LBW and normal birth weight (NBW) piglets supplemented with different concentrations of dietary Trp (NBW with 0% Trp, N0; LBW with 0% Trp, L0; LBW with 0.4% Trp, L4; LBW with 0.8% Trp, L8; *N* = 6 in each group.) and conducted systematic, transcriptome-wide analysis using RNA sequencing (RNA-seq). We identified 39 differentially expressed genes (DEG) between N0 and L0, and genes within “increased dose effect” clusters based on dose-series expression profile analysis, enriched in fatty acid response of gene ontology (GO) biological process (BP). We then identified RNA-binding proteins including *SRSF1*, *DAZAP1*, *PUM2*, *PCBP3*, *IGF2BP2*, and *IGF2BP3* significantly (*P* < 0.05) enriched in alternative splicing events (ASE) in comparison with L0 as control. There were significant positive and negative relationships between candidate genes from co-expression networks (including PID1, ANKRD44, RUSC1, and CYP2J34) and postprandial plasma TG concentration. Further, we determined whether these candidate hub genes were also significantly associated with metabolic and cardiovascular traits in humans via human phenome-wide association study (Phe-WAS), and analysis of mammalian orthologs suggests a functional conservation between human and pig. Our work demonstrates that transcriptomic changes during dietary Trp supplementation in LBW piglets. We detected candidate genes and related BP that may play roles on lipid metabolism restoration. These findings will help to better understand the amino acid support in LBW metabolic complications.

## Teaser

Dietary l-tryptophan supplementation can rescue lipid metabolism through regulating fatty acid response related genes in low birth weight piglet model.

## Introduction

Low birth weight (LBW) is a result of 2 main restrictions: insufficient gestational age (preterm) and/or distressed fetal environment (Goldenberg and Culhane, 2007). In addition to association with increased neonatal and infant mortality, some clinically chronic diseases including cardiovascular, metabolic, renal, and brain-based disorders are prevalently considered to be related to LBW (Barker, 2007; Abel et al., 2010; Buhl et al., 2018). LBW is also reported to trigger “developmental origins of adult disease” (de Boo and Harding, 2006), particularly metabolic syndrome detected in young adults, with tissue- and gene-specific alterations in epigenetic levels (Meaney et al., 2007). In livestock production, some deteriorative effects of LBW, such as hindgut epithelial barrier dysfunction, immature digestive systems, and lower fiber number in skeletal muscle (Michiels et al., 2013; Sciascia et al., 2019; Tao et al., 2019), impose a burden on husbandry production.

As a key regulator of systemic energy homeostasis, liver balances glucose and lipid metabolism through the mediation of hepatokines response (Jensen-Cody and Potthoff, 2021). Liver can serve as an endocrine organ to achieve inter-organ communication with distant tissues, to avoid metabolic dysregulation caused by chronic liver diseases including insulin resistance and/or fatty liver disease (Jensen-Cody and Potthoff, 2021). Previous studies identified bio-indicators that connect LBW and metabolic diseases. For example, increasing plasma leptin level happened in LBW children and adiponectin concentration is positively correlated with birth weight and BMI (Kotani et al., 2004; Miras et al., 2010); higher risk of diabetes and obesity is statistically verified when focusing on middle age people with LBW (Jornayvaz et al., 2016). Little is known about transcriptome changes in low and normal birth weight (NBW) neonates relative to our understanding of mechanisms underpinning LBW phenotype and correlated adult disease susceptibility.

A proper amino acid nutrition formula has been demonstrated to mitigate animal and human LBW consequences caused by intrauterine growth restriction during gestation (Lin et al., 2014; Wang et al., 2014b; Weckman et al., 2019), while few studies have investigated rescuing postnatal LBW phenotype. As one of 9 essential amino acids supplied by diet, increasing l-tryptophan (Trp) supplementation serves an essential role on fetal growth and development (Ligam et al., 2005), through the Trp catabolism-produced derivatives, such as serotonin and melatonin (Taleb, 2019). Trp intake potentially connects the link between intestinal microbiota regulation and cardiometabolic health (Tang et al., 2017), producing a possible strategy to rescue postnatal LBW by nutritional supplementation. In newborn piglets’ livers, differentially expressed genes (DEG) involved in excess Trp supplementation compared with control are associated with NADH oxidation, reactive oxygen species (ROS) metabolism and tissue development, reflecting the metabolic importance of Trp in energy consumption and homeostasis (Xu et al., 2019). Previously we reported that Trp supplementation can mitigate postprandial plasma triglycerides (TG) concentrations compared with LBW controls (Goodarzi et al., 2021). Therefore, it is of great interest to detect changes in the transcriptome and biological processes (BP) in LBW after Trp supplementation.

In this study, we set out to identify global gene regulatory mechanisms, DEG, and underlying pathways that shed further light on possible relevancy and mechanisms of growth after typtophan supplementation as observed by Goodarzi et al. (2021). Transcriptome-wide analysis including differentially expressed genes, co-expression network, RNA-binding protein enrichment in alternative splicing events (ASE), and correlation between candidate genes and physiological index of plasma TG was conducted. Furthermore, phenome-wide association analysis (Phe-WAS) based on human database was performed to confirm functional conservation of hub genes. The results are identification of key hub genes that may be responsible for more global changes in gene regulation and candidate pathways.

## Materials and Methods

Experimental procedures used in this study were in accordance with the FASS Guide for the Care and Use of Agricultural Animals in Research and Teaching and were approved by Oklahoma State University’s Institutional Animal Care and Use Committee (Animal Care and Use Protocol-IACUC-19-71).

### Animals and sample collection

Liver tissue samples used in this study were collected as part of our previous study, where specific details about animals, housing, and tissue collection can be found (Goodarzi et al., 2021). Briefly, we selected 7-d-old male piglets from 12 sows with similar parity and litter size. One NBW (>1 kg) piglet and 3 LBW piglets (<1 kg) were selected from each sow. In addition to basal milk replacement, piglets were assigned into 4 groups and supplemented with different amounts of Trp: (1) NBW piglets without Trp supplementation (N0, *n* = 8); (2) LBW piglets without Trp supplementation (L0, *n* = 8); (3) LBW piglets with 0.4% Trp supplementation (L4, *n* = 7); (4) LBW piglets with 0.8% Trp supplementation (L8, *n* = 8). These 7-d-old piglets had 3 d of adaptation and experimental diets started from day 3. Body weight was measured before starting the study and during the experimental days (days 3 to 21). At the end of study (day 21), 2 h after meal the piglets were euthanized using the CO2 asphyxiation method. From each group, 6 piglets were randomly chosen and liver tissues collected, flash frozen in liquid nitrogen and stored at −80 °C for further analysis.

### mRNA extraction for RNA-seq

We extracted total mRNA from 24 liver tissues using a RNAzol RT reagent (Sigma-Aldrich, St. Louis, MO, USA) according to the manufacturer’s procedure. Collected RNA samples were purified using AMPure XP system, and quality was assessed by Agilent Bioanalyzer 2100 system. All samples passed the quality control with the concentration ranged from 116 to 1,349 ng/μL, and the RNA integrity numbers were between 7.9 and 9.6, which met the requirement for transcriptome sequencing. cDNA library preparation and RNA sequencing were performed by Novogene Co Ltd. (CA, USA), generating paired-end reads at 150 bp length on the Illumina NovaSeq 6000 System. We obtained an average of 21.5 million read pairs per samples, which ranged from 19.7 to 23.3 million across 24 samples. Raw sequencing data reads are deposited in FASTQ format to the NCBI Sequence Read Archive database under the BioProject accession number PRJNA828586.

### Transcriptome assembly and quantification

Prior to assembly, adapters were trimmed and quality assessed for raw reads using Trim Galore wrapper scripts v0.6.5 (https://www.bioinformatics.babraham.ac.uk/projects/trim_galore/). Reads with low-quality base calls (phred score <= 20) were removed. Preprocessed *fastq* files were aligned to the *Sus Scrofa11.1* reference genome (Warr et al., 2020) using Hisat2 (v2.2.1) (Kim et al., 2019) with default parameters and SAM files were transformed to sorted BAM format by SAMtools v1.6.0 (Li et al., 2009). Transcriptional coordinates and expression values were generated as individual GTF files using Stringtie v2.1.4 (Kovaka et al., 2019).

### Detection of DEG

Transcript expression was exported as read count matrices from GTF files and differential expression analysis was performed by edgeR v3.32.1 after *filterByExpr* function was used to remove low expressed genes. The genes with false discover rate (FDR; adjusted *P*-value) < 0.1 were considered differentially expressed.

### Gene ontology analysis

The enriched BP in gene ontology terms (GO) was identified using goseq R package (Young et al., 2010), which requires genes length information to take length bias into account (Oshlack and Wakefield, 2009). BP with overrepresented *P* < 0.01 were regarded as enriched.

### Dose-series expression profile analysis

Expression data of DEG in L0 vs. N0 comparison were normalized using the transcripts per million (TPM) method (Conesa et al., 2016) and base mean values among replicates in four groups (L0, L4, L8, and N0) were used for further scale and normalization, in which values in L0 were centered to 0. The genes’ relationship was calculated via *Minkowski* distance and clustered into 3 groups based on expression trends responding to the dosage of Trp in LBW piglets.

### Co-expression network construction

The correlation coefficients among genes differentially expressed in L0 vs. N0 were evaluated performing Pearson correlation method in R program via normalized TPM expression. Base mean values of TPM from 6 biological replicates at each treatment were utilized for calculation. The network degree for each gene was determined by the number of other genes that show high correlation (correlation coefficients > 0.9 (positive) or < −0.9 (negative)) with the corresponding gene and these genes were highlighted if the number of highly correlated genes is more than 15. These correlations were put into Cytoscape software (v3.7.2) to visualize co-expression networks.

### Identification of alternative splicing events

To compare ASE among diverse Trp supplementations, we utilized rMATs (v4.1.0), a statistical model for detection of differential alternative splicing from replicate RNA-Seq data (Shen et al., 2014). We used rMATs with default parameter and inclusion level of alternatively spliced exon in different ASE, including skipped exon (SE), alternative 5ʹ splice site (A5SS), alternative 3ʹ splice site (A3SS), mutually exclusive exons (MXE), and retained intron (RI) events were come out. The percent of exon inclusion (read counts of spliced exon included/(read counts of spliced exon included + read counts of spliced exon excluded)) in L0 group was compared with other groups and difference of percent spliced inclusion (ΔPSI) was used for determining differential ASE. In each comparison with L0 group as the control, the splicing events with *FDR* adjusted *P* < 0.1 and |ΔPSI| ≥ 0.05 were detected differentially spliced.

### Motif enrichment analysis of RNA-binding proteins

To identify the RNA-binding proteins (RBP) enriched in transcripts which leads to differential ASE between L0 and other groups, we both collected known motifs of RBP and performed motif scan by RBPmap web server (Paz et al., 2014). The binding regions of RBP were determined using the similar steps as previous research (Lin et al., 2016). Binding regions were set including alternatively spliced exon body and corresponding flanking sequences that are 250 bp upstream or downstream of this exon. Ten base pairs upstream of the 5ʹ splice site and 10 bp downstream of the 3ʹ splice site in flanking regions were excluded. Enrichment analysis was performed using RBP-related motifs and binding regions to detect the frequency of motifs being paired and not paired with binding regions. As controls, binding regions from non-differential ASE were randomly selected as the background. Fisher’s exact test (one-sided test) was performed to identify significantly enriched motifs in binding regions of differential ASE with *P* ≤ 0.05. Only motif with the smallest *P*--value in each RBP was remained when there were multiple motifs in one RBP.

### Sequence conservation analysis

To determine the sequence conservation of genes that showed top network degrees in the co-expression network, the gene conservation scores (Gene Orthologs) were calculated by averaging the percentage of the *Sus scrofa* sequence matching the sequence of other 7 mammals (including human, mouse, blue whale, dog, horse, sheep, and cow). The conservation scores of specific genes across 7 different mammalian species compared with pig were determined by the percentage of corresponding mammal sequence matching the pig sequence.

## Results

### Trp has no effect on growth rate of LBW piglets

**Figure 1.**
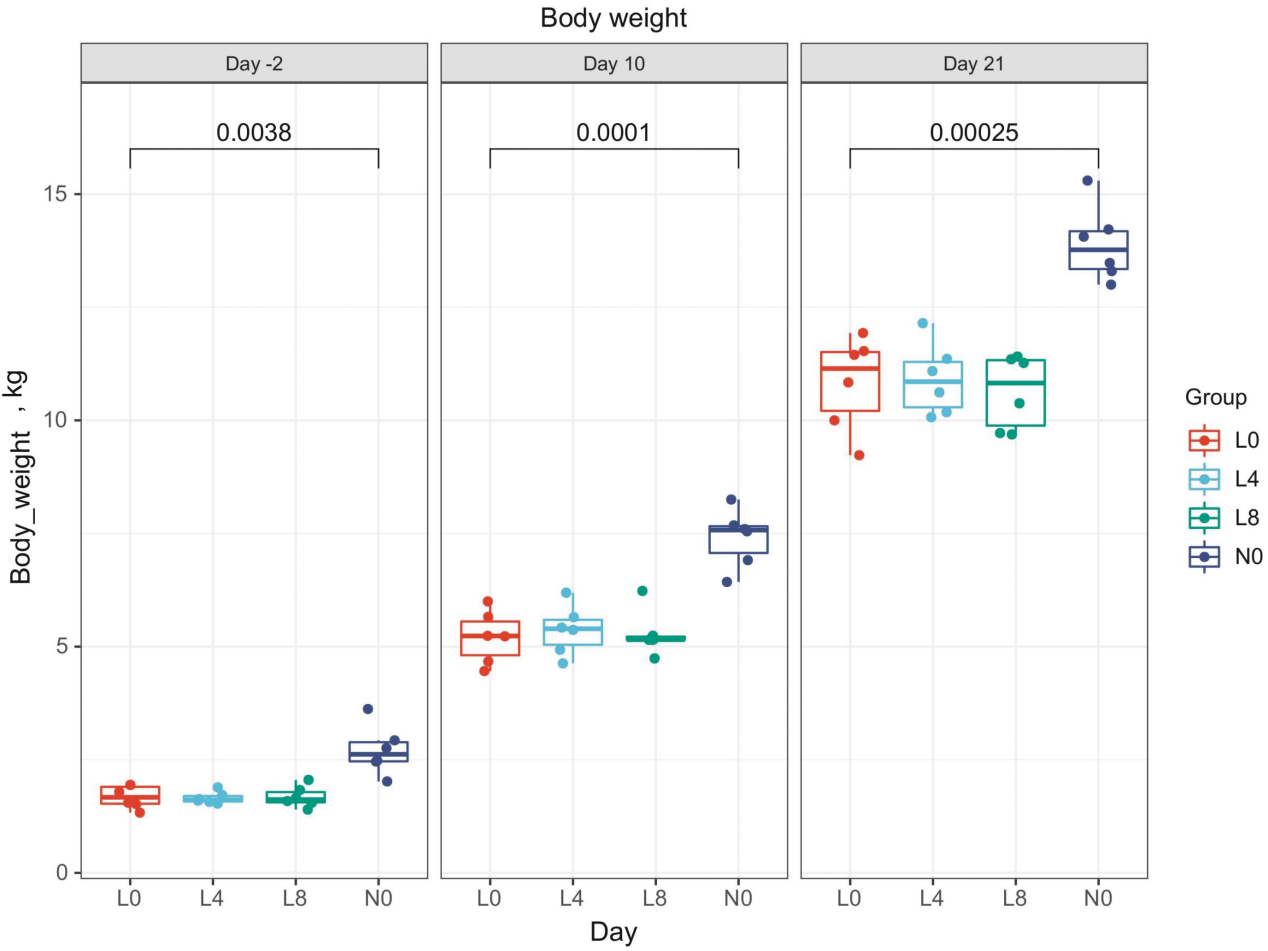
Growth tendency of 4 groups of piglets (kg). All 7-d-old piglets had an adaptation from days 0 to 3, and experimental diets were supplied from day 4.

Piglets were classified into NBW and LBW groups based on the initial birth weight criterion (1 kg) and we investigated whether Trp supplementation could ameliorate the growth rate of LBW piglets. There was no significant difference of the body weight in L0 (1.63 ± 0.24 kg) vs. L4 (1.66 ± 0.13 kg) or L0 vs. L8 (1.68 ± 0.23 kg) at 5 d preceding introduction of the experimental diets (day −2), while there was obvious weight difference between L0 and N0 (2.55 ± 0.40 kg, *P* = 0.0038). After 19 d of Trp supplementation (day 21), there were still no differences in weight among L0 (10.83 ± 1.03 kg), L4 (10.91 ± 0.79 kg) and L8 (10.64 ± 0.81 kg), but a significant difference between L0 and N0 (14.22 ± 0.82 kg, *P* = 0.00025). As a result, Trp supplementation failed to rescue the growth rate of LBW piglets (Figure 1).

### Expression profiles during dietary Trp supplementation

**Figure 2.**
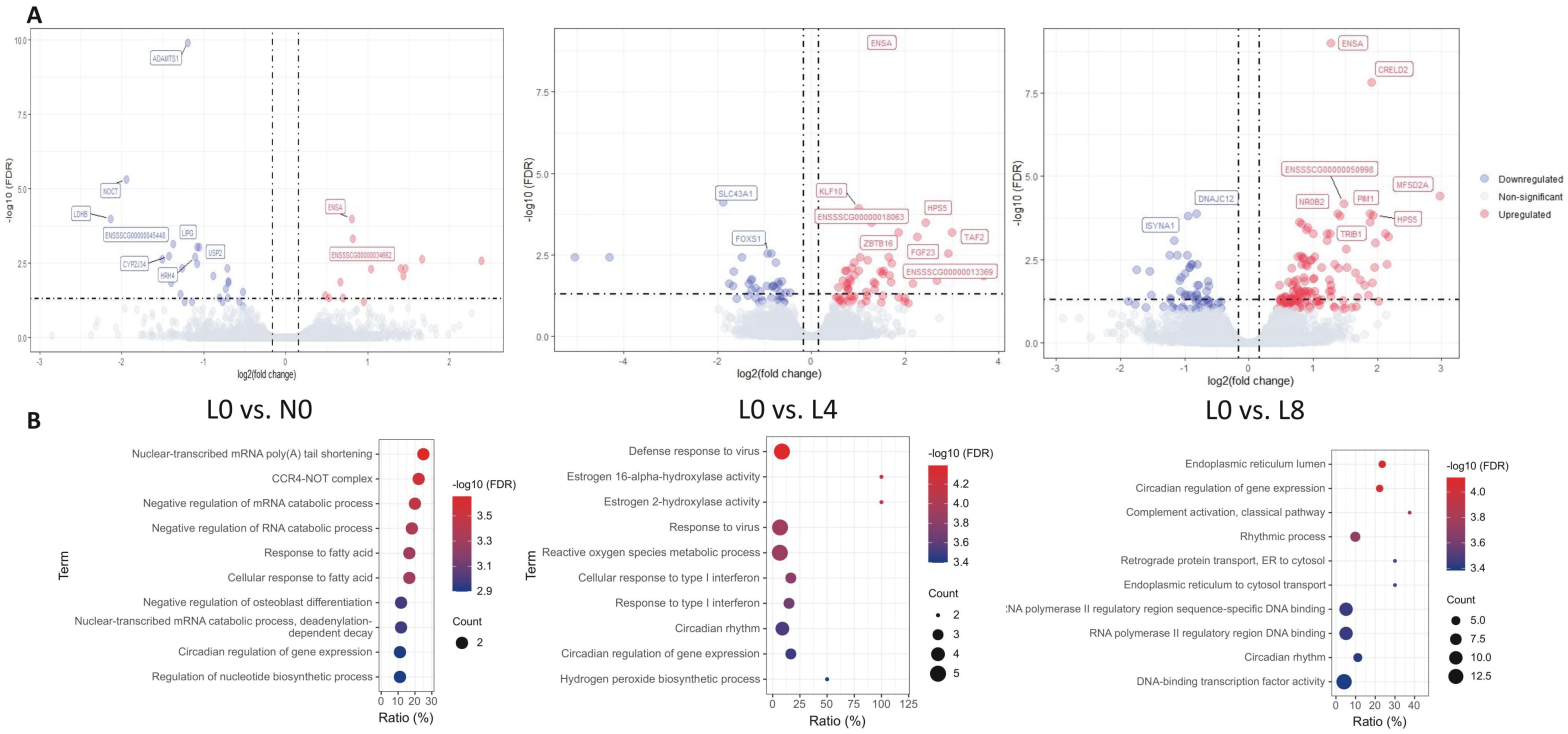
Identification of DEG and corresponding BP enrichment. (A) From top left to bottom left: volcano plots of DEG in L0 vs. N0, L0 vs. L4 and L0 vs. L8 comparisons, respectively (FDR < 0.1). Top 5 downregulated (blue) and upregulated genes are labeled. (B) Enrichment of BP in GO analysis of 3 comparisons (*P* < 0.01).

After the quality control of raw RNA-seq data (in Methods), the average mapping rate of 24 samples from 4 groups (L0, L4, L8, and N0) was 95% (ranging from 93% to 97%). According to the default cutoff of filterByExpr function in the edgeR package (Robinson et al., 2010), 15,344 genes out of 30,477 Ensembl annotated genes were retained after filtering low expressed genes. There were 100, 191, and 39 DEG in L4, L8 and N0 groups compared with L0 (Figure 2A)Supplementary file. The role of DEG in each pairwise comparison was identified through GO enrichment analysis. LBW piglets with Trp supplementation had significant differences in ontology enrichment for viral immune response mechanisms and synthesized protein transportation (Figure 2B) compared with control. Interestingly, DEG in L4 and L8 as well as NBW pigs were all enriched in circadian rhythms, which are important BP in fatty acid response and glucose homeostasis (Shi et al., 2019).

### Gene clustering and co-expression network based on LBW characteristics

**Figure 3.**
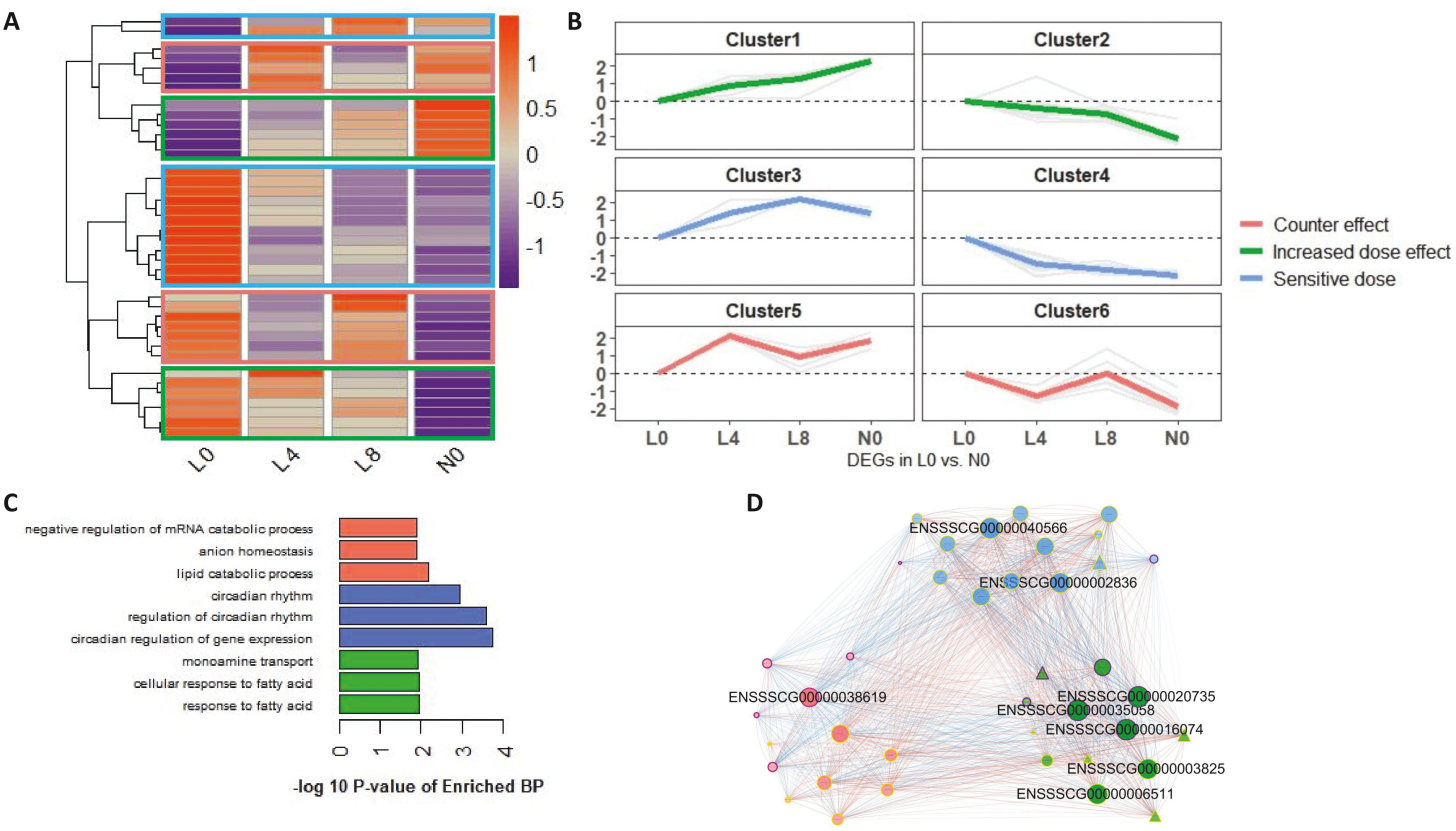
Gene characteristics of DEG in L0 vs. N0. (A) Heatmap of DEG clustering based on complete linkage clustering algorithm. (B) Three categories of expression profiles of DEG based on the response to Trp dose effects. (C) BP enrichment of 3 DEG clusters (*P* < 0.01). GO terms in red, blue, and green represent the results from “Counter effect”, “Sensitive dose”, and “Increased dose effect” clusters, respectively. (D) Co-expression network based on Pearson correlation. Notes with purple and yellow circles represent DEG upregulated and downregulated in N0, respectively. Red and blue edges show the positive and negative relation between DEG, respectively.

Since many DEG in L4 an L8 were unique to DEG in N0 (Supplementary file), we only used N0 DEG to perform gene clustering and reflect their tendencies in Trp supplementation groups. Six gene clusters were identified using complete linkage clustering algorithm with normalized expression of 39 DEG (Figure 3A). These clusters were termed into 3 expression profiles by their expressional trends going through the L0, L4, L8, and N0 sequence, including “Increased dose effect”, “Sensitive dose”, and “Counter effect” profiles having 13, 14, and 12 genes, respectively (Figure 3B). Corresponding enrichment analysis revealed genes in the “Increased dose effect” group were significantly enriched in BP associated with fatty acid response, while the “Sensitive dose” and the “Counter effect” groups were enriched in BP associated with circadian rhythm and catabolic processes, respectively (Figure 3C). Pearson’s correlation coefficient among 3 expression profiles was evaluated, and genes having more pairs with strong correlation (|*r*| > 0.9) were considered hub genes. The top 20% of genes ranked by strong correlation were highlighted in Figure 3D, with 5, 2, and 1 hub genes remaining in their respective group. Combined with enrichment results, hub genes involved in “Increased dose effect” had strong correlations with known metabolism-related genes. For instance, one DEG, pyruvate dehydrogenase kinases 4 (*PDK4*, ENSSSCG00000015334) had strong co-expression coefficients with *PAFAH2* (ENSSSCG00000038619, *r* = −0.905055843) and *N4BP2L1* (ENSSSCG00000040566, *r* = 0.956966535). As a hub gene, phosphotyrosine interaction domain containing 1 (PID1) significantly correlated with *PAFAH2* (ENSSSCG00000038619, *r* = 0.949663082), *MAP3K15* (ENSSSCG00000020735, *r* = 0.983973896), *ANKRD44* (ENSSSCG00000016074, *r* = 0.998611358), *CYP2J34* (ENSSSCG00000003825, *r* = −0.960171041), *N4BP2L1* (ENSSSCG00000040566, *r* = −0.974340728), *RUSC1* (ENSSSCG00000006511, *r* = −0.978620047), and *SALL1* (ENSSSCG00000002836, *r* = −0.987612628) (Figure 3D; Supplementary file).

### Identification of RNA-binding proteins regulating the LBW-associated alternative splicing events

**Figure 4.**
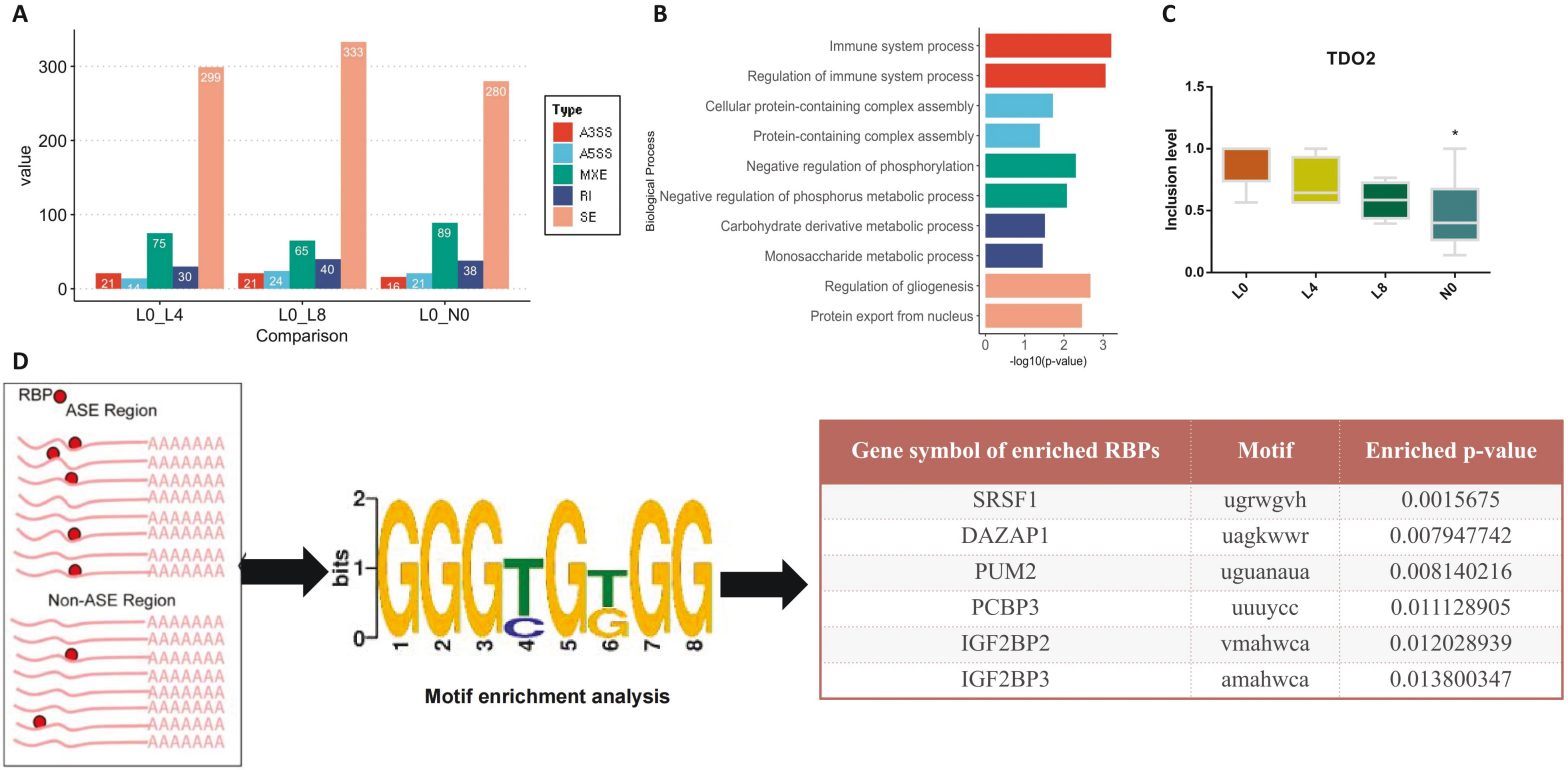
Identification of RBP regulating the LBW-associated ASE. (A) The count of ASE in 5 splicing patterns when L0 was control among 3 comparisons (FDR < 0.1). (B) Biological process enrichment of ASE overlapped genes in L0 vs. N0 (*P* < 0.05). C. Boxplot of percent spliced inclusion (PSI) level of TDO2 among 4 groups, * means *P* < 0.05 between L0 and N0. D. Motif enrichment analysis of LBW-associated RBP. RBP with *P* < 0.05 were considered as enriched in Fisher’s exact test.

Previous studies demonstrated post-transcriptional RNA splicing is spatiotemporally specific in metabolic processes (Nikolaou et al., 2019; Goodson et al., 2020; Yu et al., 2020). We identified those differentially ASE between L0 and other groups in order to explore a possible relationship between mRNA splicing and metabolic regulation. ASE among four groups were characterized by applying rMATs (Shen et al., 2014) and using L0 as the control to compare with others. Among three comparison groups, there are a similar number of splicing patterns (L0 vs. L4, L8, and N0, respectively, Supplementary file; Figure 4A). Notably, SE constitutes most alternative splicing patterns in all 3 groups (Figure 4A). Genes containing ASE between L0 and N0 were significantly enriched in metabolic and immune system processes (Figure 4B), which agree with reports of susceptibility to disease and metabolic dysfunction (Endo-Umeda et al., 2012; Bruijnesteijn et al., 2018; Zhang et al., 2020). Intriguingly, tryptophan 2,3-dioxygenase (TDO2), a gene participating Trp catabolism through the kynurenine pathway (Badawy, 2020), showed a significantly low SE inclusion level in the NBW group compared to L0 piglets, and low inclusion levels were also observed after the supplementation of Trp in LBW groups (L4 and L8) (Figure 4C).

As RBP regulate RNA splicing (Dreyfuss et al., 2002), we further investigated the enrichment feature of RBP among different birth weight groups. Splicing events in L0 were considered as control, and significantly differential splicing events in all 3 comparisons were defined as Trp metabolism-associated differential splicing events in LBW. In total, 10 A5SS, 7 A3SS, 88 SE, 17 MXE, and 8 RI events were included in RBP enrichment analysis (Supplementary file). The information of RBP’s RNA-binding motif and regions where alternatively spliced exon body happened in ASE (see methods) were employed to identify RBP-binding enrichment in ASE. As a result, RBP including *SRSF1*, *DAZAP1*, *PUM2*, *PCBP3*, *IGF2BP2*, and *IGF2BP3* were significantly enriched in co-existed ASE compared with alternatively spliced exon body in non-ASE (Figure 4D).

### LBW related genes may regain function via Trp supplementation to achieve lipid metabolism restoration

**Figure 5.**
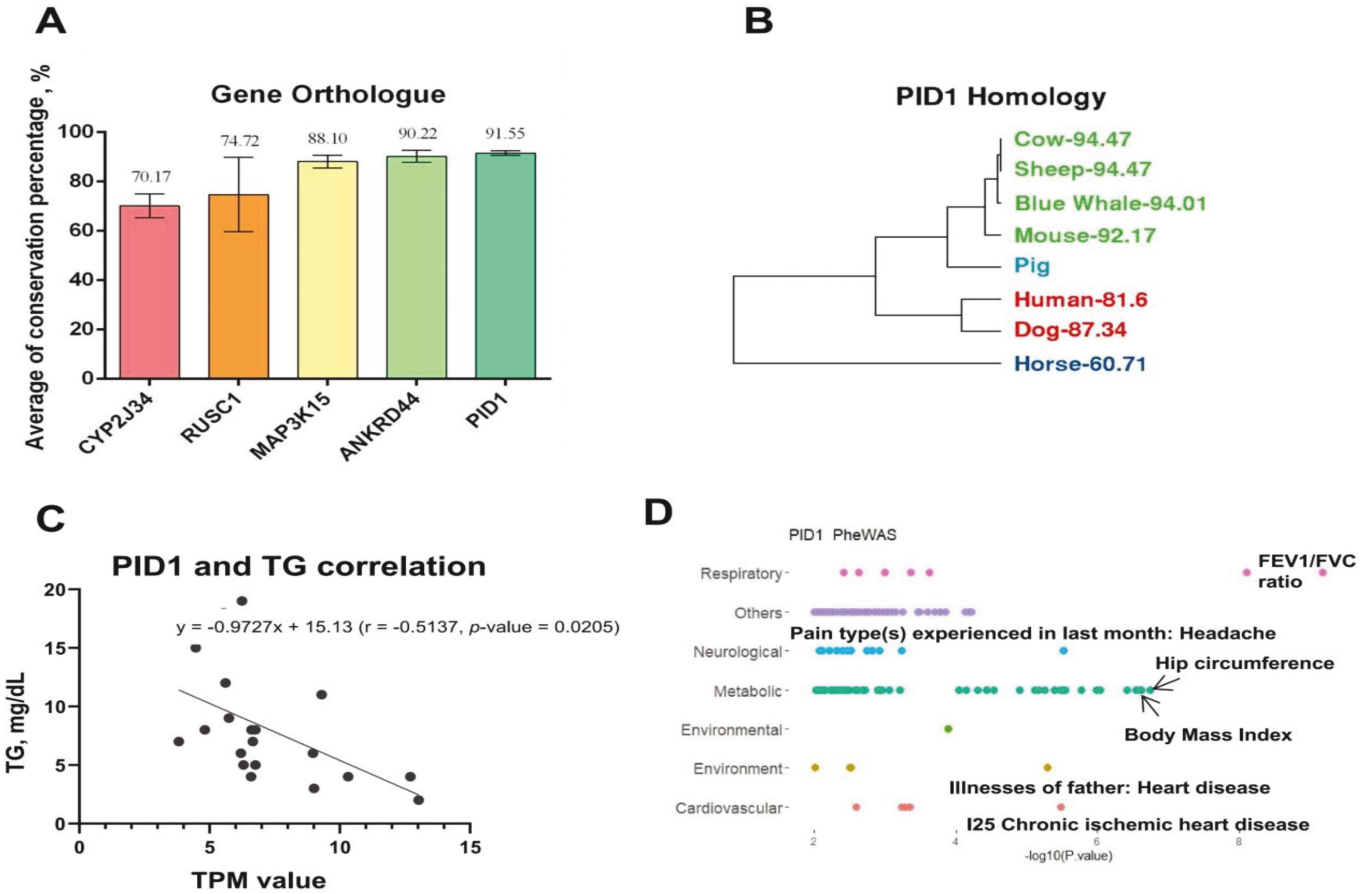
PID1 may play a role in metabolic recovery by Trp supplementation. (A) The bar-plot shows the top 5 averaged gene conservation scores of 5 hub genes among seven mammalian species. (B) Hierarchical tree of 7 mammalian species based on the conservation scores of PID1 compared with Sus scrofa. (C) Scatter plot shows the correlation (*r*) between expression levels (TPM) of PID1 and levels of postprandial plasma TG (mg/dL) across 4 groups. (D) Phe-WAS results for PID1, where *P*-values are determined by the *t*-test between metabolic traits and the corresponding types of traits (*P* < 0.01).

In order to investigate whether candidate genes of lipid metabolism function similarly in mammalian species, we performed a 7-mammalian ortholog comparison of these genes. Five lipid metabolism-related DEG were highly conserved (>70%) among seven mammals (Figure 5A). As an example, we show sequence conservation of PID1 among 7 mammals and swine (Figure 5B). PID1 was upregulated in the NBW group and increased expression in LBW piglets given Trp dosage augmentation (Figure 5C). Interestingly, plasma TG levels and PID1 expression displayed a negative correlation among samples in 4 groups (Figure 5D), indicating an effective TG reduction with lipid metabolism-related genes activation after supplementing Trp in LBW groups. We also found that TG showed both positive and negative relationships with other candidate genes exhibiting similar expression profiles between Trp supplemented and NBW groups (Supplementary Figure S3). To understand the potential function of hub genes, we conducted Phe-WAS analysis using the human orthologs of these candidate genes with 3302 unique traits in different domains (https://atlas.ctglab.nl/). We found PID1 as well as other LBW-related hub genes were significantly associated (*P* < 0.01) with metabolic and cardiovascular traits (Figures 5E; Supplementary Figure S4), suggesting vital roles on lipid metabolism regulation and chronic effects on cardiovascular health in mammals (Liu et al., 2015; Aliwarga et al., 2018; Li et al., 2020).

## Discussion

LBW is not only associated with neonatal mortality and morbidity due to the metabolic defects (Wang et al., 2008, 2018; Huang et al., 2021), but also leads to adverse postnatal growth with chronic health threats (Sangild et al., 2006; Dziaman et al., 2007). In recent years nutritional support strategies have been suggested to aid in achieving catch-up growth in LBW infants and animal models. LBW piglets have been widely used in these studies due to the similarity in structure, development and metabolic process with human metabolic organ system (Ferenc et al., 2014). Overnutrition compensation in LBW individuals may cause excessive lipid accumulation, obesity, type II diabetes, and hypertension due to the LBW-specific insulin-resistance state (Dulloo, 2006; Dulloo et al., 2006). Therefore, it is necessary to identify methods that rescue metabolic routine and potentially prevent lifelong disease. Trp is an essential amino acid that synthesizes serotonin (5-hydroxytryptamine), a neurotransmitter that regulates feeding behavior and body weight (Lam et al., 2010). Previously we reported that Trp supplementation in LBW piglets can significantly affect plasma TG concentration and lipid metabolism markers such as acetyl-CoA carboxylase α and hydroxyacyl-CoA dehydrogenase (Goodarzi et al., 2021). Elevated Trp in daily diet decreased serum TG levels and have serum uric acid-lowering effects to efficiently prevent hyperuricemia in human (Oshima et al., 2019). In our study, we utilized DEG from L0 vs. N0 comparison to perform genes clustering, aiming to uncover genes that show expression profiles similar to N0 in Trp-supplemented LBW groups (L4 and L8). Genes within “Increased dose effect” were enriched in fatty acid response process, which is in line with decreased plasma TG levels in Trp supplied groups (Goodarzi et al., 2021). Identifying gene clusters via correlation-based co-expression networks strengthen our understanding of gene coordinated regulation.

It has been reported that mRNA splicing plays roles in lipid metabolism of multiple species (Medina et al., 2008; Gingras et al., 2014; Lv et al., 2019). ASE in liver biopsies of obese patients were associated with metabolic comorbidities and RNA-binding proteins such as *SFRS10*, *SFRS7*, and *HNRPA1* were significantly decreased in liver of obese subjects (Pihlajamaki et al., 2011). Since the degree of intron retention will affect the pre-mRNA maturing and its functional integrity (Zheng et al., 2020), we identified the ASE among 4 groups to determine whether they were LBW-related ASE. We found TDO2 showed higher SE inclusion level in L0, while this value was decreased and approached the N0 in Trp supplemented groups. To investigate the causal factors of ASE, we conducted the motif enrichment of RBP, which serve as sensors to perform pre-mRNA splicing when they interact with core spliceosome within ASE regions (Fredericks et al., 2015). Based on the specified regions within ASE and non-ASE (as random controls), similar to previous research (Lin et al., 2016), we determined enriched motifs and corresponding RBP by Fisher’s exact test. For example, serine/arginine-rich splicing factor 1 (*SRSF1*) has been reported to regulate liver tumor growth rate, and loss of body weight in mice (Wang et al., 2014a). Insulin-like growth factor 2 mRNA-binding protein 2 (*IGF2BP2*) is considered as genetic loci related to increased threat to diabetes type 2, as well as participating in lipid metabolism and insulin production (Tarnowski et al., 2019).

We described candidate hub genes in our co-expression networks by identifying genes with the greatest number of significant associations between themselves and other genes. As one of the hub genes, *PID1* was reported to increase lipolysis through inhibiting AKT/PKA pathway in adipose tissue of mice (Yin et al., 2019). Mu et al. (2019) also proposed that overexpressed *PID1* in pig’s liver can reduce the serum high-density lipoprotein cholesterol (HDL-C) level. There were significant positive and negative relationships observed between candidate genes and corresponding plasma TG levels, demonstrating the consistent results that hepatic *PID1* mediates postprandial lipoproteins and plasma TG disposal (Fischer et al., 2018). Therefore, we propose *PID1* may be an important gene in lipid metabolism and recover plasma lipid absorption in Trp-supplemented LBW piglets. Nevertheless, it is of value to collect additional RNA-seq data and multiple bio-indicators in later growth periods to correlate gene expression and metabolic dynamics with the effect of Trp supplementation in LBW individuals.

## Conclusions

In this study, we investigated the impact of dietary Trp supplementation on the ability to rescue the LBW caused metabolic abnormality using RNA-seq data and bio-indicators. Our results provide novel insights into molecular mechanisms underlying Trp supplementation and metabolic recovery in LBW piglets, highlighting the potentials of supplying Trp to counter inborn metabolic disorders in human. The results presented here provide candidate genes, pathways, and putative molecular mechanisms for further study.

## Supplementary Material

skac156_suppl_Supplementary_Materials

## Abbreviations

A3SS: alternative 3ʹ splice site
A5SS: alternative 5ʹ splice site
ASE: alternative splicing event
BP: biological process
DEG: differentially expressed gene
FDR: false discover rate
GO: gene ontology
HDL-C: high-density lipoprotein cholesterol
IGF2BP2: insulin-like growth factor 2 mRNA-binding protein 2
L0: low birth weight without dietary tryptophan supplementation
L4: low birth weight with 0.4% dietary tryptophan supplementation
L8: low birth weight with 0.8% dietary tryptophan supplementation
LBW: low birth weight
MXE: mutually exclusive exons
N0: normal birth weight without dietary tryptophan supplementation
NBW: normal birth weight
Phe-WAS: phenome-wide association study
PID1: phosphotyrosine interaction domain containing 1
RBP: RNA binding protein
RI: retained intron
RNA-seq: RNA sequencing
ROS: reactive oxygen species
SE: skipped exon
SRSF1: serine/arginine-rich splicing factor 1
TDO2: tryptophan 2,3-dioxygenase
TG: triglyceride
TPM: transcripts per million
Trp: l-tryptophan
