# Supplementary figures and images for: RNA-seq reveals insights into molecular mechanisms of metabolic restoration via tryptophan supplementation in low birth weight piglet model
Source: J Anim Sci. 2022 May 11;100(5):skac156. doi: 10.1093/jas/skac156 (PMC9155244; doi:10.1093/jas/skac156)

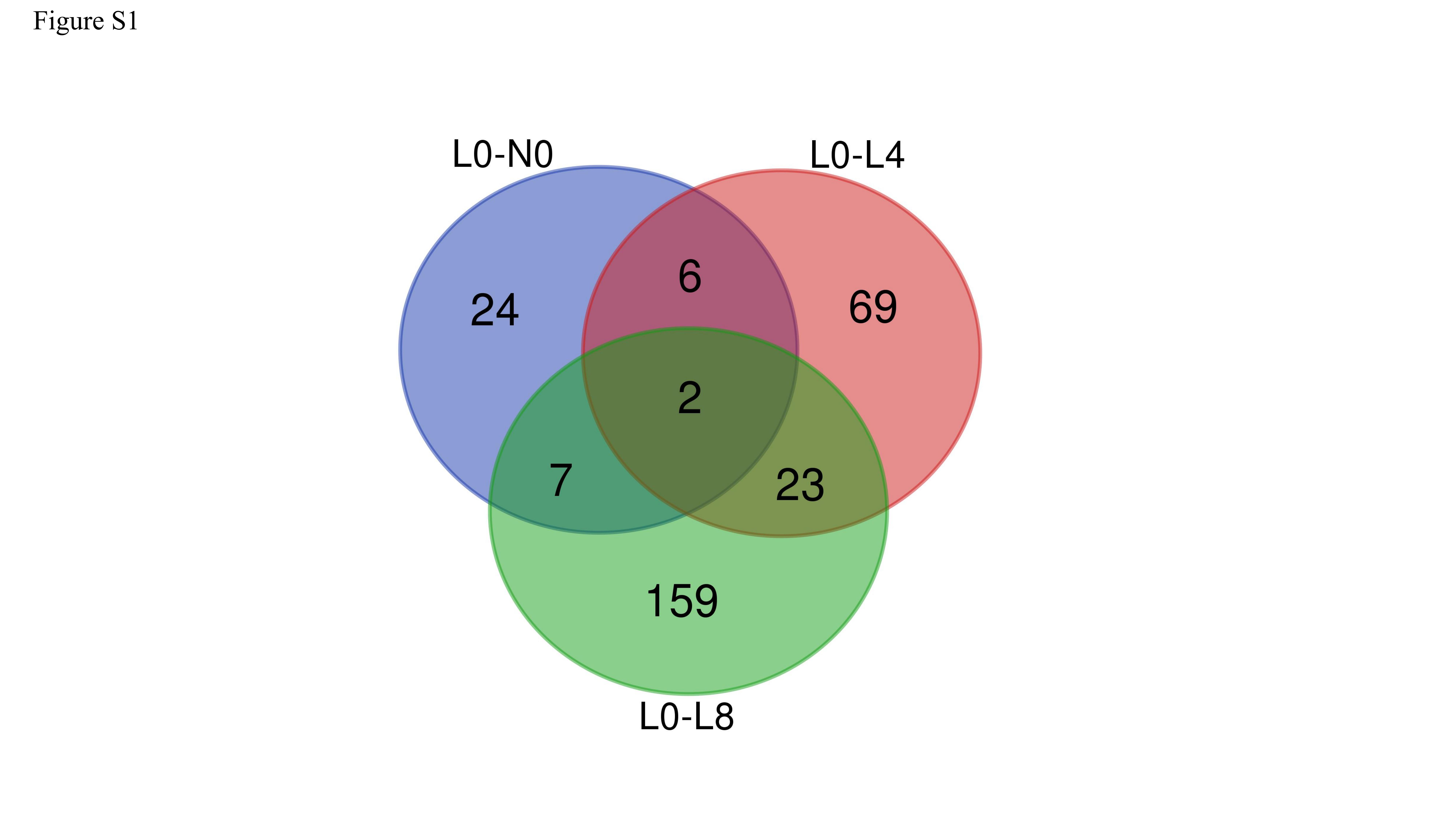

Supplement: skac156_suppl_Supplementary_Materials [file skac156_suppl_supplementary_materials.zip › skac156_suppl_Supplementary_Figure_S1.jpeg]

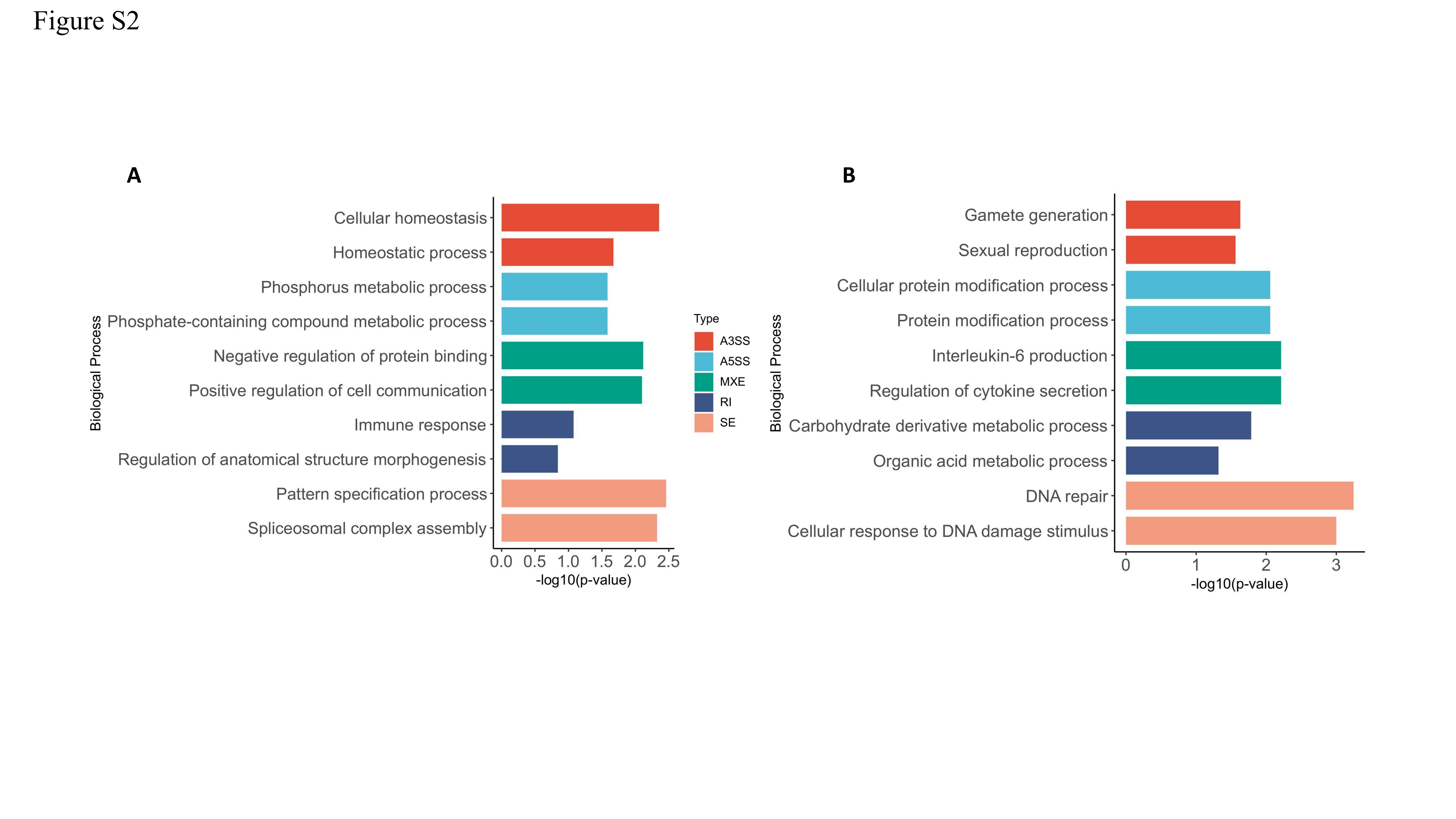

Supplement: skac156_suppl_Supplementary_Materials [file skac156_suppl_supplementary_materials.zip › skac156_suppl_Supplementary_Figure_S2.jpeg]

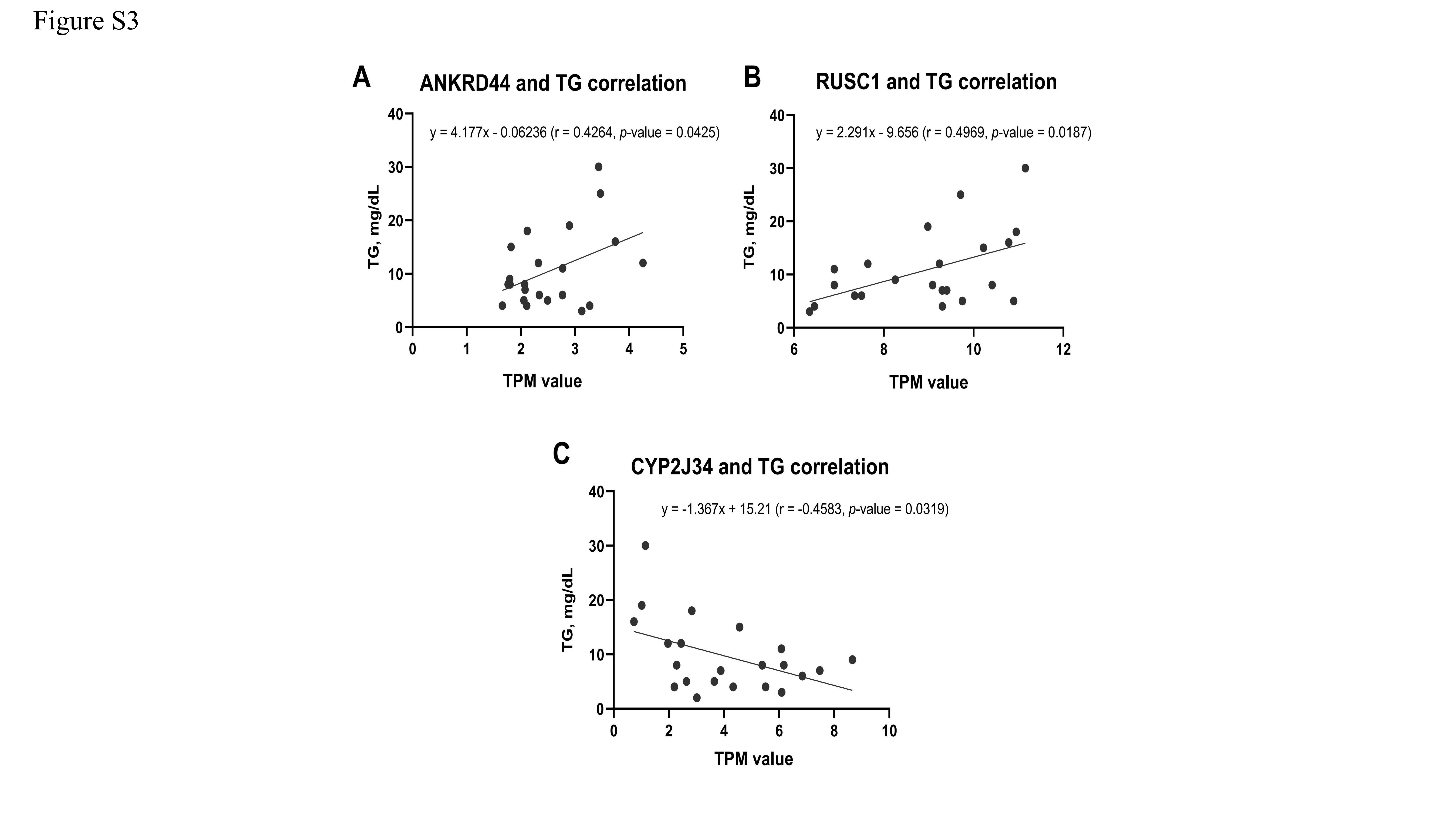

Supplement: skac156_suppl_Supplementary_Materials [file skac156_suppl_supplementary_materials.zip › skac156_suppl_Supplementary_Figure_S3.jpeg]

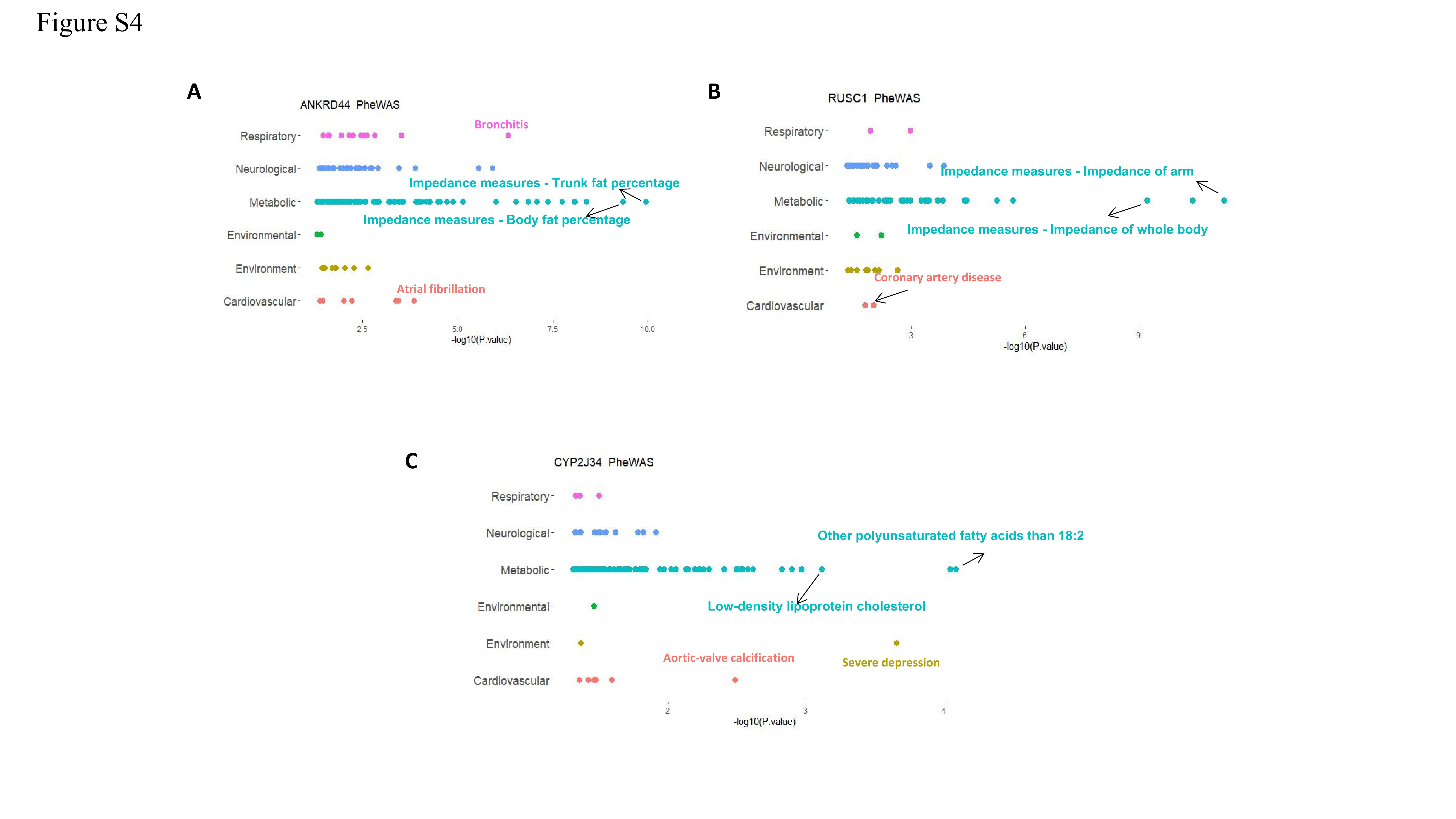

Supplement: skac156_suppl_Supplementary_Materials [file skac156_suppl_supplementary_materials.zip › skac156_suppl_Supplementary_Figure_S4.jpeg]
